# High-Performance Surface Electromyography Armband Design for Gesture Recognition

**DOI:** 10.3390/s23104940

**Published:** 2023-05-21

**Authors:** Ruihao Zhang, Yingping Hong, Huixin Zhang, Lizhi Dang, Yunze Li

**Affiliations:** School of Instrument and Electronics, North University of China, Taiyuan 030051, China

**Keywords:** wearable device, acquisition system, surface electromyography (sEMG) signal, convolutional neural networks (CNNs), gesture recognition

## Abstract

Wearable surface electromyography (sEMG) signal-acquisition devices have considerable potential for medical applications. Signals obtained from sEMG armbands can be used to identify a person’s intentions using machine learning. However, the performance and recognition capabilities of commercially available sEMG armbands are generally limited. This paper presents the design of a wireless high-performance sEMG armband (hereinafter referred to as the α Armband), which has 16 channels and a 16-bit analog-to-digital converter and can reach 2000 samples per second per channel (adjustable) with a bandwidth of 0.1–20 kHz (adjustable). The α Armband can configure parameters and interact with sEMG data through low-power Bluetooth. We collected sEMG data from the forearms of 30 subjects using the α Armband and extracted three different image samples from the time–frequency domain for training and testing convolutional neural networks. The average recognition accuracy for 10 hand gestures was as high as 98.6%, indicating that the α Armband is highly practical and robust, with excellent development potential.

## 1. Introduction

Surface electromyography (sEMG) signals reflect the comprehensive effects of surface muscle and nerve trunk electrical activity on the skin. sEMG data can be acquired noninvasively through simple operations [1]. Different muscle activities and muscle states produce sEMG signals with varying amplitude and frequency, which can reflect the activity of the muscles. Therefore, sEMG has been widely used in the monitoring and evaluation of muscle conditions, prosthetic control, motion posture recognition, and human–machine interfaces [2] and has attracted considerable attention in industry.

Since the introduction of the Myo Gesture Control Armband by Thalmic Labs, the market for sEMG data-acquisition systems has grown rapidly. However, the Myo Armband is limited by its eight channels, 8-bit analog-to-digital converter (ADC) resolution, 200 sps sampling rate, and bandwidth of 5–100 Hz. The Biomedical Microsystems Laboratory at Laval University designed a 3DC Armband, which had the following improved device parameters: a 10-bit ADC resolution, 1000 sps sampling rate, and bandwidth of 20–500 Hz. Under the same experimental conditions, the 3DC Armband significantly outperformed the Myo Armband [3], demonstrating the necessity of increasing the number of channels, sampling rate, and resolution. However, the use of a 10-bit ADC to acquire data transmitted in 16-bit format resulted in the wastage of system resources.

Phinyomark et al. compared sEMG recognition results obtained with sampling rates of 200 and 1000 sps and found that the 200-sps sampling rate significantly reduced the amount of discriminatory information used for sEMG control, and the difference in frequency information features significantly affected the classification results. The accuracy of recognizing multiple hand and finger movements was significantly increased by high−frequency components, particularly for individuals who had undergone radial artery amputation, with differences exceeding 10% [4]. In addition, the test results of reference [5] also indicate that the sampling rate will affect the prediction accuracy of gestures.

In summary, the performance of wearable sEMG data-acquisition devices needs to be improved. In this study, we designed a 16-channel sEMG Armband with wireless communication, a 16-bit ADC resolution, an adjustable bandwidth (0.1–20 kHz), and an adjustable sampling rate with a maximum value of 2000 sps/channel. According to the Nyquist–Shannon sampling theorem, this device can effectively acquire sEMG signals up to 1000 Hz. The high sampling rate allows the accurate identification and classification of sEMG signals through close approximation of the original waveform [6]. The adjustable bandwidth and sampling rate of the device allow sEMG signals to be flexibly acquired by filtering the frequency bands that the user is not interested in and adapting the sampling rate to different working conditions.

## 2. Hardware Design

The surface electrodes at the front-end of the sEMG acquisition system convert the ionic currents generated by human electrochemical activity into electronic currents, which are detectable by the electronic detection system [7]. Wet electrodes often require the use of electrolyte gel, which can stimulate the human skin, and can be inconvenient to use. Thus, they are usually replaced with dry electrodes [8,9]. In our design, customized 8 mm × 6 mm × 1 mm gold-plated copper electrodes were used, with one sEMG channel corresponding to three electrodes: a pair of differential electrodes and a reference electrode. There were 16 channels and 48 electrodes in total.

RHD2216 (Intan Technologies, Los Angeles, CA, USA) is a low-power physiological signal-acquisition chip with dimensions of only 8 mm × 8 mm. It features 2.4 µVrms input reference noise, a 16-bit ADC, and 16 differential channels. The amplifier bandwidth can be changed by configuring the registers, and the amplifier offset can be eliminated by an internal digital high-pass filter. The 16-channel differential inputs connect to the differential electrodes and the surface EMG signals are collected in parallel and sent to the microcontroller unit (MCU) in real time through the serial peripheral interface.

STM32F765VIH6 is a high-performance controller in the ARM Cortex-M7 series from STMicroelectronics (Geneva, Switzerland). It has a digital signal processor (DSP), a floating-point unit (FPU), and an adaptive real-time (ART) accelerator, with a maximum clock frequency of 216 MHz and high-speed data-processing capabilities. In addition, it uses thin fine pitch ball grid array (TFBGA) packaging, with dimensions of only 8 mm × 8 mm. Because the surface of the EMG signal-acquisition system needs to contact the body surface, the system volume and weight are critical parameters. The controller is ideal for sEMG signal acquisition owing to its excellent data-processing capability and small size. The MCU continuously monitors the battery status in the idle mode and provides feedback to the user through indicator lights and motors. The MCU receives instructions from the base station through interrupts and configures the upper and lower cutoff frequencies (the lower cutoff frequency supports 0.1–500 Hz, and the upper cutoff frequency supports 100–20 kHz), the sEMG sampling rate (up to 2000 sps/channel), and the inertial measurement unit (IMU) sampling rate (up to 100 Hz), according to the instructions. The parameter configurations are stored in flash memory and are read when the device restarts to perform configuration operations. The MCU reads the values of the corresponding registers to determine whether the configuration was successful and provides feedback on the configuration results to the base station. After receiving the acquisition instructions, the MCU collects sEMG and IMU signals at the configured sampling frequency through direct memory access (DMA), until a stop instruction is received or the device’s power is insufficient. The device supports both wired and wireless transmission channels, with low-power Bluetooth for wireless communication and a universal synchronous/asynchronous receiver/transmitter (USART) for wired communication. The MCU determines the source of the instructions and replies with data on the corresponding channel.

The IMU uses ICM-20948 (InvenSense Inc., San Jose, CA, USA), which has a built-in three-axis gyroscope, three-axis accelerometer, three-axis compass, and digital motion processor (DMP). It is encapsulated in a 3 mm × 3 mm × 1 mm (24-pin quad flat no-led (QFN)) package. The system supports multiple 200-mAh lithium batteries in parallel or a Type-C power supply. BQ24075 is responsible for charging and discharging management, and the LP5912 series low-dropout regulator (LDO) chip provides 1.8- and 3.3 V voltages. BQ27220 is responsible for monitoring the battery level and other parameters. The low-power Bluetooth module HJ-185IMH with dimensions of 5.5 mm × 5 mm × 1.3 mm is used as the wireless transceiver. It can support a maximum speed of 1 Mbps. The sEMG and IMU data are received through a USB dongle (HJ-380IMH).

**Figure 1:**
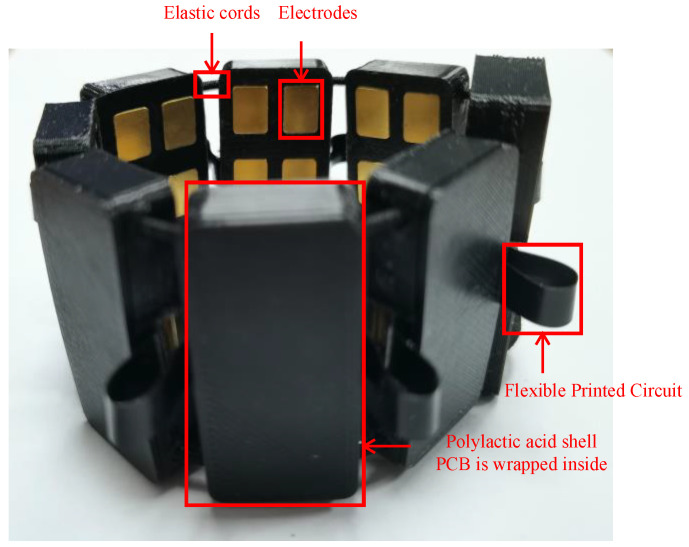
α Armband.

The α Armband is shown in Figure 1. The printed circuit board (PCB) is designed with soft and hard boards, and eight hard board areas are connected by flexible printed circuit (FPC). The equipment uses a 3D-printed polylactic acid (PLA) shell, and eight shells are connected by elastic cords. The telescopic range of the armband is 120 mm–330 mm, which ensures that it can be worn by different people. The whole armband weighs less than 100 g, and users can wear it easily.

## 3. Computer Software

**Figure 2:**
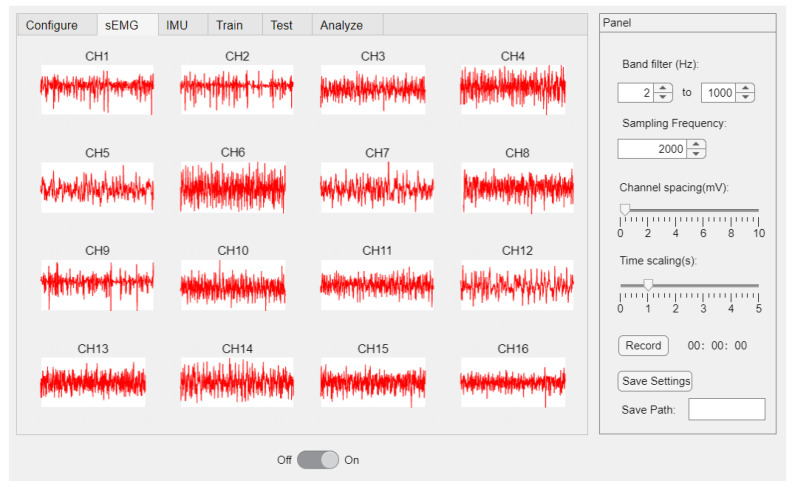
sEMG platform.

The authors designed a user-friendly software for the α Armband through MATLAB R2022a which facilitates data interaction with the hardware through the manipulation of the USB dongle. The software displays real-time sEMG waveforms of 16 channels and has functions for configuring hardware parameters and saving data (Figure 2).

## 4. Experimental Method

### 4.1. Sample Dataset

To prevent the loss of high-frequency components of sEMG data, a sampling rate of 2000 sps was used in this experiment. The upper computer software received sEMG data in real time through Bluetooth and saved the received data. Finally, the data were analyzed and processed using MATLAB.

**Figure 3:**
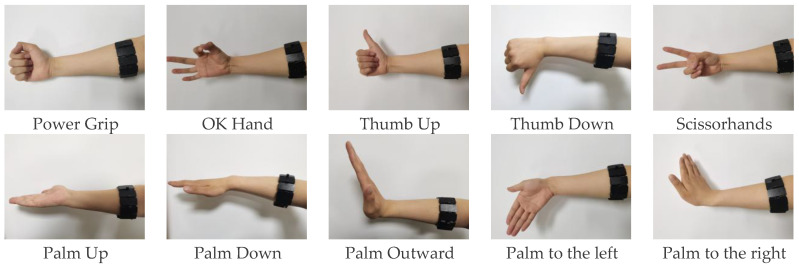
Ten gestures of a subject.

During the experiment, the α Armband was worn on the right forearm of the subject, who had rested adequately to ensure that muscle fatigue would not introduce interference. Additionally, the electrode placement area was cleaned and disinfected before the experiment. The participants were asked to perform 10 common hand gestures: Power Grip, OK Hand (in China), Thumb Up, Thumb Down, Scissorhands, Palm Up, Palm Down, Palm Outward, Palm to the left, and Palm to the right (Figure 3).

The experiment involved 30 participants, including 20 males and 10 females between the ages of 20 and 50 years. Among them, 24 were right-handed, and 6 were left-handed. Each participant was asked to maintain a gesture for 10 s, followed by a 3 s rest period, and then to repeated the same gesture 10 times. This process was repeated for all 10 gestures. The experiments lasted for a week, with one experiment conducted per day. The data used to the train machine-learning were the average values for the 10 repetitions of each gesture. All participants agreed to allow their collected sEMG data to be used free of charge for academic research purposes.

### 4.2. Preprocessing

sEMG signals are often contaminated by noise due to interference from the surrounding environment and the circuitry of the acquisition device, which can sometimes overpower the sEMG signal. To obtain the true sEMG signal, noise reduction measures must be taken [10,11,12].

On the hardware side, the cutoff frequency of the built-in high-pass filter of RHD2216 was configured to 2 Hz. The sampling frequency in the experiment was 2000 Hz. To comply with the Nyquist–Shannon sampling theorem, the cutoff frequency of the low-pass filter was configured to 1000 Hz for constructing a bandpass filter that screened out noise outside of the frequency band. The built-in custom digital module of RHD2216 was used to implement a single-pole high-pass filter on each sampling amplifier channel, with the aim of eliminating the residual direct-current (DC) offset voltage associated with the analog amplifier.

**Figure 4:**
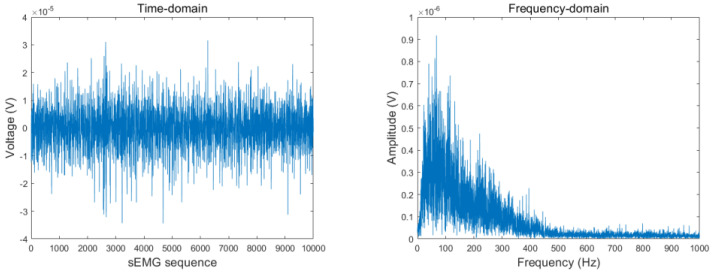
sEMG time- and frequency-domain plots (before Butterworth notch filtering).

**Figure 5:**
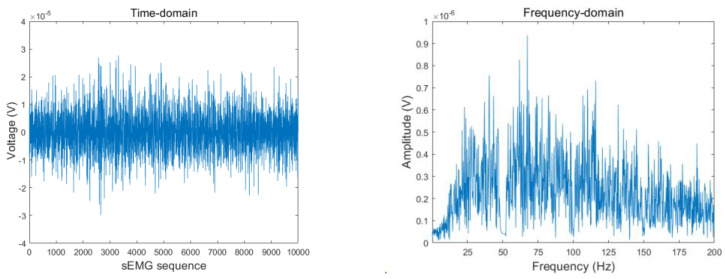
sEMG time- and frequency-domain plots (after Butterworth notch filtering).

Electric-field interference can affect sEMG detectors through capacitive coupling. In a 50-Hz alternating-current (AC) power environment (the rated frequency of China’s state grid is 50 Hz, and the deviation is not allowed to exceed ±0.5 Hz), 50 Hz AC power can easily interfere with sEMG acquisition equipment. Poor grounding and many other factors can induce powerline frequency interference into the equipment [13]. The collected data were filtered using a 50-Hz Butterworth notch filter with a lower cutoff frequency of fc1 = 49 Hz and an upper cutoff frequency of fc2 = 51 Hz to eliminate 50 Hz power frequency interference. Figure 4 and Figure 5 present the sEMG time- and frequency-domain plots before and after filtering with the Butterworth notch filter. As shown, the 50 Hz signal was significantly attenuated.

### 4.3. Feature Extraction and Classification

High-accuracy gesture recognition is a key goal pursued by researchers. Thus, feature extraction and classification are among the most critical stages in sEMG control systems. Classification techniques based on sEMG have been extensively studied [14], and sEMG feature extraction approaches generally focus on time-domain features, frequency-domain features, or time–frequency mixed features. Time-domain features are intuitive and can clearly reflect the changes of sEMG signals over time. For instance, the peak value reaching a certain threshold can be used as the judgment criterion for a corresponding gesture, while the root-mean-square (RMS) value reflects the average level of sEMG over a period of time. Frequency-domain features help to reveal components of the signal, including the median frequency, mean power frequency, and spectral area [15,16]. Angkoon Phinyomark et al. extracted eight feature sets from the time–frequency domain and achieved a maximum accuracy of 89.7%, leaving ample room for further improvement [4].

The manual extraction of sEMG signal features often leads to significant errors. Deep learning can automatically extract features at different levels from a large number of input samples, avoiding the complex and cumbersome process of manual feature extraction and selection [17]. In recent years, researchers have used various classifiers to classify sEMG, including k-nearest neighbor (KNN), linear discriminant analysis (LDA), and support vector machines (SVMs), etc. [18,19]. Deep learning has been widely applied in computer vision. sEMG data from electrodes can be transformed into image data through calculations, and then features can be extracted from the images [20]. Convolutional neural networks (CNNs) significantly reduce the number of network parameters via local connections and weight sharing, making the training easier. In the pooling layer, downsampling further reduces the number of training parameters of the network, reduces the impact of pixel value changes on the convolution results, and improves the generalization ability of the network. Therefore, CNNs have better robustness and intelligence in processing images [21,22,23]. Duan Na et al. [24] showed through comparative experiments that CNNs have higher gesture recognition accuracies than SVMs.

In our experiment, two comparative methods were designed on the basis of the CNN architecture. The data of 8 sEMG channels of the α Armband were used as one group (distributed evenly across 8 channels) and the data of 16 sEMG channels were used as the other group to examine the effect of the number of channels on the gesture recognition accuracy. The collected sEMG data were divided into three types according to the time–frequency domain: time-domain image inputs, multi-channel spectral image inputs, and feature-enhanced multi-channel spectral image inputs.

### 4.4. Time-Domain Image Inputs

For real-time wearable sEMG devices, the total response time of myoelectric control should be limited to 300 ms [25,26,27]. A wide window function bandwidth results in a low time-domain resolution, and a narrow window function bandwidth results in a low frequency-domain resolution [28]. To ensure real-time performance, 250 ms of sEMG data for each channel was segmented using a 100-ms sliding window with an increment of 50 ms. The resulting sEMG data from 16 channels were converted into an image of size 16 × 250, and those from 8 channels were converted into an image of size 8 × 250, as the input for the CNN.

**Figure 6:**
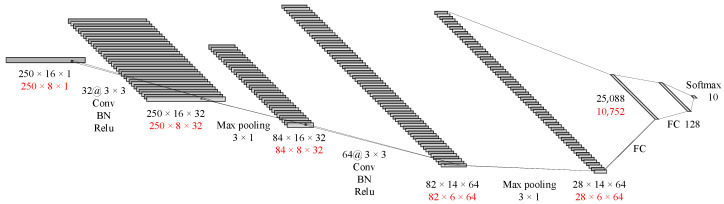
CNN for time-domain images (black represents 16 channels; red represents 8 channels).

As shown in Figure 6, the first layer of the CNN is a convolutional layer with 32 kernels, each having a size of 3 × 3 and a stride of 1. To better preserve edge information, a layer of 0 padding is added around the input matrix. This layer also includes batch normalization and the rectified linear unit (ReLU) activation function, which are mainly used to extract shallow features. The second layer is a 3 × 1 max pooling layer. The next convolutional layer has 64 kernels, each with a size of 3 × 3 and a stride of 1, and no padding is used. This layer also includes batch normalization and the ReLU activation function. The next layer is a 2 × 1 max pooling layer, followed by two fully connected layers and finally the softmax output.

### 4.5. Multi-Channel Spectral Image Inputs

**Figure 7:**
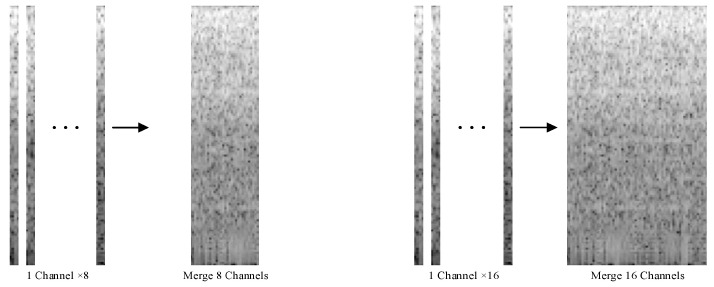
Stitching of 8- and 16-channel spectral images (Three dots represent the omitted single−channel image, and the arrow points to the synthesized image).

In this experiment, a sliding window of the Hann function was used to perform the short-time Fourier transform (STFT) on sEMG signals for acquiring the spectral features of the signals. A 100 ms window with a 50 ms increment was used to segment a 250 ms sEMG signal, resulting in a 4 × 129 output matrix. As shown in Figure 7, the 8- and 16-channel output matrices were concatenated to obtain 32 × 129 and 64 × 129 matrices, respectively, which corresponded to 32 × 129 and 64 × 129 grayscale images used as inputs for the CNN.

**Figure 8:**
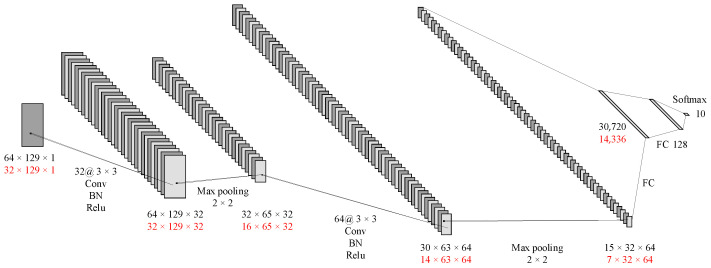
CNN for spectral images (black represents 16 channels; red represents 8 channels).

The CNN architecture for the multi-channel spectral images was similar to that of the time-domain images. Some parameters were adjusted according to the input images. As shown in Figure 8, The first layer has 32 convolution kernels, each with a size of 3 × 3 and a stride of 1, and was padded with a layer of 0. It includes batch normalization and the ReLU activation function. The second layer was a 2 × 2 max pooling layer. The next convolutional layer has 64 kernels, each with a size of 3 × 3, a stride of 1, and no padding. It also includes batch normalization and the ReLU activation function. The fourth layer was a 2 × 2 max pooling layer, followed by two fully connected layers. Ultimately, the result was output through the softmax function.

### 4.6. Feature-Enhanced Multi-Channel Spectral Image Inputs

**Figure 9:**
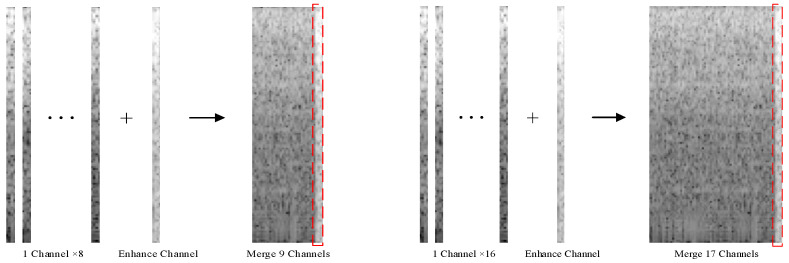
Stitching of 8- and 16-channel feature-enhanced spectral images(Three dots represent the omitted single−channel image, and the arrow points to the synthesized image. The dotted red box represents the enhanced channel image).

**Figure 10:**
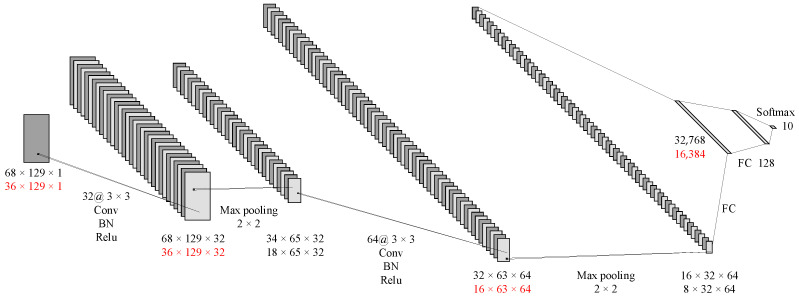
CNN for feature-enhanced spectral images (black represents 16 channels; red represents 8 channels).

To enhance the feature representation of the signal, Duan Na et al. used the Myo Armband to extract the maximum value of the sEMG signal from each channel as an auxiliary channel signal. They merged the spectrograms of the eight original channels and one auxiliary channel into a single image as the input of the CNN. The test results indicated that after multiple iterations, the accuracy of the CNN reached 94.06% [24]. In this study, we extracted the maximum values from the 8- and 16-channel signals of the α Armband as auxiliary channels, forming 9-channel and 17-channel sEMG signals (Figure 9), respectively. To highlight the comparison effect, we segmented the signals using the method presented in Section 4.5 and performed the STFT, without changing the CNN architecture (Figure 10).

## 5. Test Results

**Figure 11:**
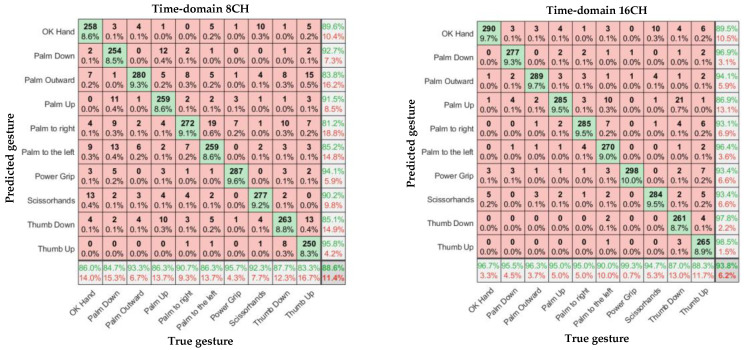
Confusion matrices of 8- and 16-channel time-domain images.

**Figure 12:**
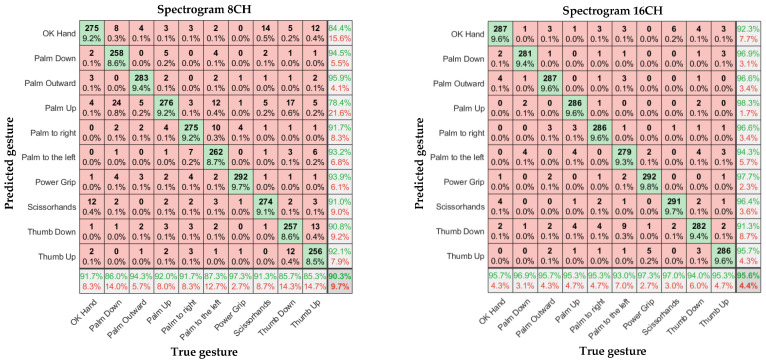
Confusion matrices of 8- and 16-channel spectral images.

**Figure 13:**
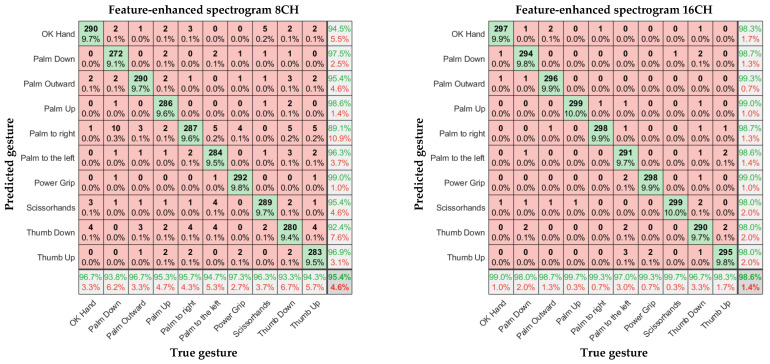
Confusion matrices of 8- and 16-channel feature-enhanced spectral images.

In Figure 11, Figure 12 and Figure 13, the left and right subgraphs show the confusion matrices of 8- and 16-channel images, respectively. The horizontal axis represents the true gesture, and the vertical axis represents the predicted gesture.

## 6. Discussion

According to Table 1, Overall, the sEMG data collected by the α Armband were highly reliable, and a relatively high gesture recognition accuracy was achieved without using complex CNN models. There were significant differences in recognition accuracy among different input samples, with an average accuracy of 88.6%–98.6% for gesture recognition.

Considering the input model, for the eight-channel experiments, the average accuracies of the time-domain image model and spectral image model were 88.6% and 90.3%, respectively. However, the average accuracy of the feature-enhanced spectral image model was 95.4%, which was 6.8% and 5.1% higher than those of the previous two models, respectively. For the 16-channel experiments, the average accuracies of the time-domain image model and spectral image model were 93.9% and 95.9%, respectively. However, the feature-enhanced spectral image model achieved an average accuracy of 98.6%, which was 4.7% and 2.7% higher than those of the previous two models, respectively. The lowest recognition accuracy of the 16-channel feature-enhanced spectral image model was 96.7%, and the highest recognition accuracy was 99.7%, which was almost perfect. The results indicate that the spectral images carry more prominent features than time-domain images and that extracting the maximum value as an auxiliary channel from the α Armband’s sEMG channels is feasible and helps the CNN extract important features more accurately.

Regarding the number of channels, in Ulysse Côté-Allard et al.’s comparative study, the 3DC Armband (10 channels, 10 bit, 1000 sps) outperformed the Myo Armband (8 channels, 8 bit, 200 sps) in all gesture recognition experiments. The average accuracies of the 3DC Armband and Myo Armband were 89.47% and 86.41%, respectively [3]. Meanwhile, the average accuracy of the α Armband (16 channels, 16 bit, 2000 sps) exceeded 95%. Thus, the number of channels, resolution, and sampling rate affect the gesture recognition accuracy. According to our experimental results, the 16-channel accuracy was significantly higher than the 8-channel accuracy. When the number of channels increased from 8 to 16, the average accuracy of the time-domain image model increased by 5.2%, that of the spectral image model increased by 5.3%, and that of the feature-enhanced spectral image model increased by 3.2%. These models had identical sampling rates, resolutions, and bandwidths, indicating that the number of channels significantly affected the gesture recognition accuracy, possibly because a larger number of channels are more likely to cover the forearm muscle group responsible for each gesture. The human forearm has six muscle groups controlling different hand movements, and each gesture involves only one part of the forearm muscle group [29,30].

In our future work, we will persist in improving the α armband, such by enabling it to detect deep muscles, because deep muscles and surface muscles work together for motion control [31,32]. However, we hope to avoid invasive testing, so we will attempt to integrate ultrasound modules into the arm loop to achieve a non-invasive detection of deep muscles. We believe that this work will improve the accuracy of gesture recognition.

## 7. Conclusions

The α Armband designed in this study has more data-acquisition channels, a higher sampling rate, and a larger bandwidth than traditional sEMG armbands. These parameters can be easily configured through Bluetooth, making the α Armband highly adaptable to various working conditions. Our experiment involving the α Armband confirmed its high reliability; it achieved an average recognition accuracy of 98.6% under the feature-enhanced spectral image model. Additionally, our experiment indicated the following:

(1) Feature-enhanced spectral images enhanced the CNN’s gesture recognition accuracy by at least 3% compared with conventional spectral images;
(2) The 16-channel sampling increased the CNN’s gesture recognition accuracy by at least 3.2%.

In future research, the authors will test a wider variety of gestures, finger movements, and limb movements and develop more feature extraction and classifier models, facilitating the application of the α Armband in a wider range of medical working conditions.

## Figures and Tables

**Table 1:** Test accuracy results.

Label	Time-Domain Diagram 8CH	Time-Domain Diagram 16CH	Spectrogram 8CH	Spectrogram 16CH	Feature-Enhanced Spectral Images 8CH	Feature-Enhanced Spectral Images 16CH
Power Grip	95.7%	99.3%	97.3%	97.3%	97.3%	99.3%
OK Hand	86.0%	96.7%	91.7%	95.7%	96.7%	99.0%
Thumb Up	83.3%	88.3%	85.3%	95.3%	94.3%	98.3%
Thumb Down	87.7%	87.0%	85.7%	94.0%	93.3%	96.7%
Scissorhands	92.3%	94.7%	91.3%	97.0%	96.3%	99.7%
Palm Up	86.3%	95.0%	92.0%	95.3%	95.3%	99.7%
Palm Down	84.7%	95.5%	86.0%	96.9%	93.8%	98.0%
Palm Outward	93.3%	96.3%	94.3%	95.7%	96.7%	98.7%
Palm to the left	86.3%	90.0%	87.3%	93.0%	94.7%	97.0%
Palm to the right	90.7%	95.0%	91.7%	95.3%	95.7%	99.3%
Average	88.6%	93.8%	90.3%	95.6%	95.4%	98.6%

## Data Availability

Data will be made available on request.
